# Wrinkled Photonic Elastomers with Dynamic Structural Color Patterns for Multilevel Optical Anti-Counterfeiting

**DOI:** 10.3390/gels12050356

**Published:** 2026-04-23

**Authors:** Xiaoqian Jiang, Pengjia Yan, Caiyun Wu, Junpeng Ke, Wenxiu Hou, Jingran Huang, Zhengzheng Lian, Ting Lü, Ling Bai

**Affiliations:** 1 School of Materials Science and Engineering, Jiangsu University, Zhenjiang 212013, China; jiang13914561903@163.com (X.J.); yanyanyan2351@163.com (P.Y.); 13974394541@163.com (C.W.); kejunpeng2023@163.com (J.K.); hou19707202859@163.com (W.H.); jrh@zua.edu.cn (J.H.); qq1700782024@163.com (Z.L.); 2 Institute of Environmental Materials and Applications, College of Materials and Environmental Engineering, Hangzhou Dianzi University, Hangzhou 310018, China

**Keywords:** structural color pattern, wrinkled photonic elastomer, anti-counterfeiting, mechanochromic, grafting

## Abstract

Structural colors generated by interference, diffraction, or light scattering offer vivid visual effects without dyes or electronic components, making them promising for flexible optical sensing. This work reports a simple stretch–plasma–release (S-P-R) strategy to fabricate wrinkled photonic elastomers (WPEs). The flexible periodic structures exhibit mechanically responsive structural colors, as tensile strain alters the grating period, generating optical signals that can be visualized and quantified by spectroscopy. The wrinkle period is tunable in the range of 0.4–3.42 μm by adjusting plasma power, exposure time, pre-stretch ratio, and film thickness. A dumbbell-shaped substrate design reduces edge-induced stress concentration. It shows improved wrinkle uniformity, with the coefficient of variation reduced from 6.64% to 2.74%, and experimental colors agreeing well with modified Bragg condition predictions. The reflection peak shows a significant shift from 356 nm to 658 nm with varying viewing angles. Patterned plasma treatment enables the selective generation of wrinkled structures, producing bright color patterns. The structural color can be fully erased at a critical strain of 20% and recovered upon release, remaining stable over multiple loading–unloading cycles. With excellent mechanical compliance and optical tunability, these materials are well-suited for integration with hydrogel-based systems and show promise for wearable devices, security marking, and anti-counterfeiting applications.

## 1. Introduction

Structural colors [1,2] in nature arise from the interaction between light and periodic micro/nanostructures, producing iridescent, metallic, and durable visual effects that are difficult to replicate. These optical phenomena enable multiple biological functions, such as predator evasion, enemy deterrence, and communication among species [3,4,5,6,7,8]. Inspired by these natural systems, researchers have developed a range of photonic structures, including multilayer films [9], photonic crystals [10,11,12,13,14,15,16], and metasurfaces [11,17,18], for sensing [19], displaying, solar cells and anti-counterfeiting applications [20,21,22,23,24]. In particular, diffraction gratings, characterized by periodic arrangements of slits or grooves, exhibit unique optical behaviors arising from light diffraction and have been widely employed in optical devices and particularly in anti-counterfeiting labeling. Their optical responses depend on the diffraction angle and groove spacing, giving rise to angle-dependent color-shifting effects [25]. The unique diffraction patterns produced by gratings are difficult to replicate, making them highly effective in preventing the counterfeiting of documents, currencies, and commercial products. Incorporating grating-based optical elements into labels or packaging or directly onto products provides distinctive, tamper-evident security features that can be easily authenticated under appropriate lighting conditions [26,27]. Furthermore, multilayer or hierarchical grating architectures can generate richer color variations and tunable optical information, thus enhancing the performance of structural-color-based security devices [28]. In addition, many organisms such as insects and chameleons can flexibly regulate their structural colors by adjusting the periodicity of nano/micro-structures in their scales or skins. Building upon these bio-inspired mechanisms, elastomer-based photonic materials have emerged as promising platforms that can convert mechanical deformation into visible optical changes. Such dynamic color-tunable systems offer exciting opportunities for advanced optical encryption and multidimensional anti-counterfeiting. For example, stretchable and mechanically actuated silicone elastomer films, inspired by the color-tunable feathers of hummingbirds, exhibit reversible color switching, excellent programmability, high stability, and long-term durability, making them suitable for applications in dynamic displays, sensors, camouflage materials, and beyond [29,30,31,32]. Furthermore, the integration of such elastomeric photonic structures with responsive hydrogels could lead to hybrid materials capable of simultaneous sensing of multiple stimuli, thereby expanding their functionality in biomedical and environmental monitoring. Beyond color-tunable photonic systems, wrinkles are widely observed in various natural systems, such as the skins of fruits, the leaves of plants [33], and the skins of animals [34,35,36,37,38,39,40]. The formation of wrinkles in these bilayer structures is primarily driven by a mismatch in mechanical properties between the layers. This classical wrinkling phenomenon arising from elastic mismatch can be precisely controlled to generate pleated gratings with well-defined geometries and complex structures, which have attracted considerable attention for applications in wettability control [41], biomimetic adhesives [42], and photonic sensors [43]. Polydimethylsiloxane (PDMS), a flexible and biocompatible elastomer with gel-like mechanical properties [44,45], provides an ideal platform for constructing such tunable wrinkle-based photonic systems.

**Figure 1:**
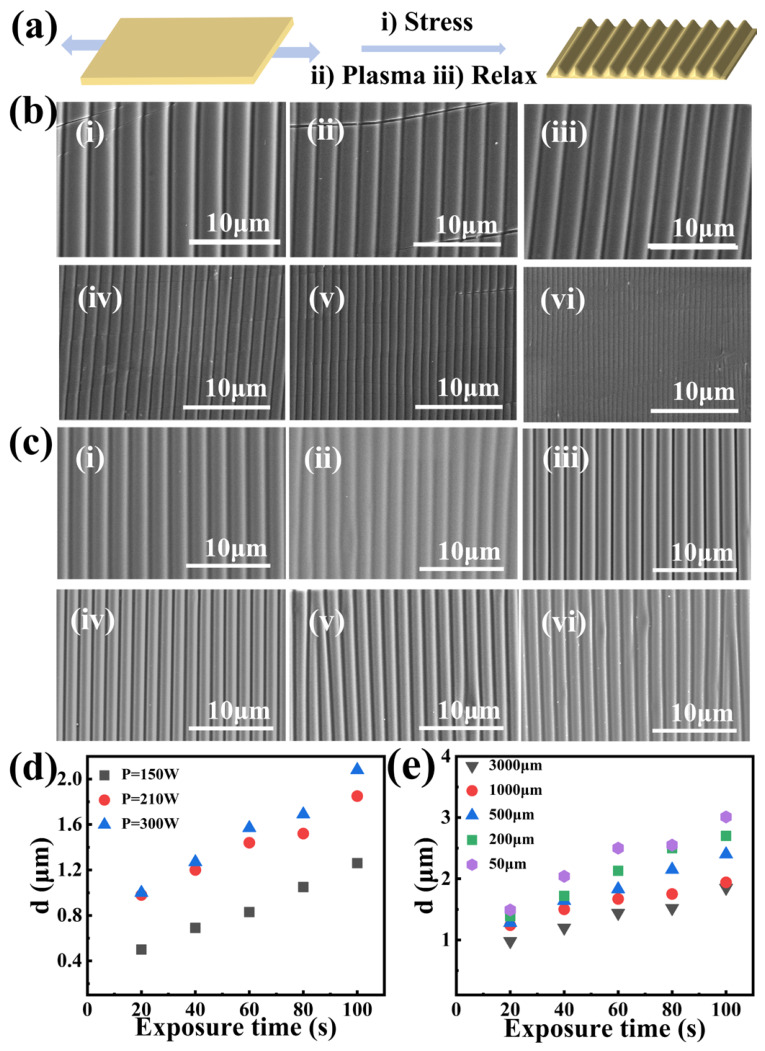
(**a**) Schematic illustration of the S-P-R strategy for fabricating WPEs. The horizontal bidirectional arrows indicate the left-right pre-stretching of the PDMS film; (**b**) SEM images of WPEs fabricated under different power levels (Preparation conditions *ε* = 10%, t = 100 s, (i) P = 300 W, (ii) P = 270 W, (iii) P = 240 W, (iv) P = 150 W, (v) P = 120 W, (vi) P = 90 W); (**c**) SEM images of WPEs fabricated under different elongation rates (Preparation conditions: t = 100 s, P = 150 W, (i) ε = 10%, (ii) *ε* = 20%, (iii) *ε* = 30%, (iv) *ε* = 40%, (v) *ε* = 50%, (vi) *ε* = 60%); (**d**) Plots showing the variation in wrinkle period with plasma power for PDMS films of thickness (h = 1000 μm); (**e**) Plots showing the variation in wrinkle period with film thickness (*ε* = 10%, P = 300 W).

Here, we utilize the variation in the elastic modulus of PDMS before and after oxygen plasma treatment to fabricate wrinkled photonic elastomers (WPEs) through a stretch–plasma treatment–release (S-P–R) strategy (Figure 1a). This straightforward approach enables the controlled fabrication of WPEs for anti-counterfeiting applications. The periods and amplitudes of the WPEs were precisely tuned by adjusting the film thickness, plasma treatment duration, and pre-stretch strain. The optical responses of the WPEs with different periodicities were further investigated under varying incident, viewing, tilt, and rotation angles, revealing distinctive and dynamic angle-dependent color-shifting characteristics. We further carried out simulations of the stress distribution of a stretched PDMS film to explain the formation of different WPEs in corresponding regions of the films, providing theoretical guidance for further optimization of S-P-R methods. Moreover, by employing cut-out templates during plasma treatment, wrinkled photonic patterns with distinct orientations and periodicities were successfully fabricated on a single PDMS substrate, endowing the system with complex optical characteristics and multiple security levels. These multifunctional photonic labels exhibit vivid structural colors and reveal visible/hidden patterns that change with variations in incident, viewing, tilt, and rotation angles or under mechanical deformation, demonstrating great potential for next-generation dynamic three-dimensional (3D) anti-counterfeiting technologies. These PDMS-based wrinkled structures also offer a promising template for transferring mechanochromic optical functionality into hydrogel platforms in future studies.

## 2. Results and Discussion

### 2.1. Fabrication and Morphological Control of Wrinkled Photonic Elastomers

Polydimethylsiloxane (PDMS) elastomer films with tunable structural colors were fabricated under various stretch–plasma treatment–release (S-P-R) conditions, resulting in surfaces with controllable wrinkle periods and amplitudes. A pre-stretched PDMS film of a defined thickness was first subjected to oxygen plasma treatment (Stretch–Plasma) [46]. During plasma exposure, the PDMS surface was oxidized to form a thin, rigid silica-like layer with a significantly higher elastic modulus than the underlying soft PDMS substrate, thereby establishing a bilayer configuration. Upon strain release, the relaxation of interfacial stress induced spontaneous surface wrinkling, producing ordered elastic wrinkles oriented perpendicular to the pre-strain direction. The effects of plasma treatment power and exposure time on the wrinkle amplitude and period were systematically investigated using this S-P-R approach. Pre-stretched PDMS folded elastomer film (*ε* = 10%) with a uniform thickness of 1 mm was treated under different plasma powers (90–300 W). The corresponding surface morphologies of the obtained WPEs are shown in Figure 1b,c. This trend is consistent with previous reports on plasma-induced wrinkling in PDMS, where higher plasma power leads to a thicker oxidized layer and consequently a larger wrinkle period [44,47]. Similarly, the decrease in wrinkle period with increasing pre-stretch ratio (Figure 1c) agrees with the classical buckling theory of bilayer systems [44]. Compared to the wrinkle periods reported by Bodas and Khan-Malek [47] (0.5–1.5 μm under similar conditions), our approach achieves a broader tunable range (0.62–3.42 μm), likely due to the wider range of plasma powers and pre-stretch ratios explored. Furthermore, recent studies by Xiong et al. [45] demonstrated that plasma-induced surface modification of PDMS creates a stiff oxynitride layer, which aligns with our mechanistic understanding of the S-P-R process. As the plasma power increased from 90 W to 300 W, the wrinkle period (d) expanded from approximately 0.62 μm to 3.42 μm. Among them, the WPEs prepared at 240 W, exhibiting a wrinkle period of 2.49 μm, were selected for further optical characterization. Using the theoretical model described in Section 4.4 (Equations (1) and (2)), the thickness of the oxidized rigid surface layer and the amplitude (A) of the wrinkled structures were calculated to be approximately 53.67 nm and 700 nm, respectively. The cross-sectional SEM image of the film (Figure S1) exhibited a nearly perfect sinusoidal profile with an experimental amplitude of 819 nm, which is in good agreement with the theoretical prediction. Theoretical analysis (see details in Section 4) predicted an amplitude of approximately 700 nm, which is in good agreement with the experimental result.

To further investigate the effects of elongation and the thickness of the amplitude and period of WPEs, the plasma power and exposure time were fixed at 150 W and 100 s, respectively. At pre-stretch ratios (*ε*) of 10%, 20%, 30%, 40%, 50%, and 60%, different elastic WPEs were obtained, as shown in Figure 1c. The wrinkle period decreased with increasing pre-stretch ratio, and a higher ε induced a greater number of cracks aligned with the stretching direction. When ε was further increased to 80%, the PDMS films fractured. Optical microscope images revealed that WPEs with large-area, periodic wrinkle structures were obtained at different elongations, with wrinkle orientation perpendicular to the transverse pre-strain direction. The structural SEM images of pre-stretched (ε = 10%) PDMS films (a cross-linked elastomer with gel-like mechanical compliance) with various thicknesses under identical plasma conditions are shown in Figure S2. The detailed results of the achievable wrinkle periods under different plasma powers, exposure times, and gel film thicknesses are summarized in Figure 1d. For PDMS films with a thickness of 1 mm and a tensile strain of 10%, the wrinkle period increased with increasing plasma power or oxidation duration, yielding an overall range of 0.4 μm to 2.0 μm. As shown in Figure 1e, when the plasma power, oxidation time, and tensile strain were fixed, the wrinkle period increased as the PDMS film thickness decreased, varying from 0.9 μm to 3.0 μm. These results collectively demonstrate that the wrinkle geometry of WPEs can be systematically tuned by adjusting plasma power, exposure time, pre-stretch strain, and film thickness, which together govern the resulting optical properties.

### 2.2. Optical Properties and Angle-Dependent Structural Colors

**Figure 2:**
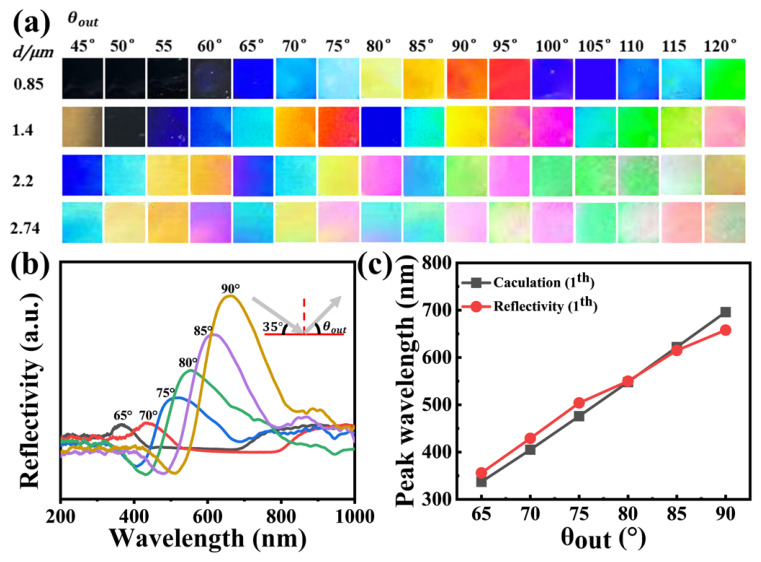
Angle-dependent color-changing performance of PDMS WPEs. (**a**) Photographs of WPEs with different wrinkle periods (d = 0.85–2.74 μm) viewed at varying observation angles (*θ_out_* = 45–120°) under a fixed incident angle (*θ_in_* = 35°); (**b**) Reflectance spectra of WPE (d = 0.85 μm) measured at *θ_in_* = 35° and *θ_out_* = 65–90°. The gray lines indicate the paths of incident light and reflected light, and the central dashed line represents the normal; (**c**) Comparison of theoretical and experimental results of the reflective peak positions of the WPEs (d = 850 nm).

To improve the structural color quality of the WPEs, we further incorporated carbon black into the wrinkles [46]. The color of the wrinkled photonic structures could be precisely tuned by varying the wrinkle periodicity and amplitude through adjustments in applied strain, plasma oxidation conditions, and other processing parameters. The vivid structural colors observed in the PDMS films can be predicted according to Bragg’s law:

(1) $$k\lambda =\;nd\left(\cos\theta_{in}-\cos\theta_{out}\right)$$

where *θ_in_* and *θ_out_* denote the angles between the incident and reflected light and the surface normal, respectively; d is the period of the WPEs; n is the effective refractive index of the surrounding medium (≈1 for air); *λ* is the reflected wavelength; and k is the diffraction order. As shown in Figure 2a, photographs of WPEs with different wrinkle periods (d = 850, 1400, 2220, and 2740 nm) were captured at varying viewing angles under a fixed incident angle of 35°.

The fabricated PDMS wrinkled films exhibited vivid structural color shifts with changing observation angle. This angle-dependent color-changing phenomenon is characteristic of diffraction gratings and has been extensively studied in both natural and synthetic photonic systems [5,7,8]. For example, the iridescent blue of Morpho butterfly wings arises from a combination of multilayer interference and grating diffraction from periodic lamellae structures [5,48], while chameleon skin color changes are mediated by tunable photonic crystal arrays [7,8]. For instance, the WPE with a period of 850 nm displayed a sapphire-blue hue at *θ_out_* = 65°, and its diffraction color gradually shifted toward longer wavelengths, changing from blue to red as *θ_out_* increased from 65° to 95°. As *θ_out_* further increased from 95° to 120°, the color of the wrinkled structure shifted back toward the blue region of the spectrum, transitioning from red to sapphire blue. Notably, this non-monotonic color-shifting behavior (blue–red–blue) differs from the monotonic redshift typically observed in conventional diffraction gratings [25,26]. Similar bidirectional color shifts have been reported in hierarchical photonic structures where multiple diffraction orders coexist [28,29]. In our WPEs, this effect arises from the interplay between first- and second-order diffraction, as confirmed by the theoretical calculations in Tables S1–S4. These observations agree well with the theoretical predictions, as summarized in Table S1. In general, increasing the viewing angle caused the second-order diffraction wavelength to shift from the ultraviolet into the visible region of the spectrum, while the first-order diffraction remained in the infrared region. The reflection peak positions of the WPE (d = 850 nm) shown in Figure 2b are in good agreement with the calculated values in Figure 2c. For WPEs with larger wrinkle periods, complex color-shifting behaviors were observed, arising from higher-order (second, third, or above) diffraction. Theoretical reflection peaks for wrinkles with periods of 1400 nm, 2200 nm, and 2740 nm are summarized in Tables S2–S4, respectively. Furthermore, when the viewing angle was fixed at 90° and the incident angle was varied, the wrinkled photonic structures exhibited distinct color-shifting appearances (Figure S3). The calculated reflection peaks (Table S5) were in good agreement with the experimentally observed colors, confirming that the color variation originated from the diffraction of gratings with different periods. The reflection spectra of PDMS WPEs (d = 850 nm) with *θ_in_* = 35° and *θ_out_* ranging from 65° to 90° are shown in Figure 2b. As the observation angle increased from 65° to 90°, the reflection peak redshifted from 356 nm to 658 nm, consistent with the color shift observed in the optical photographs (Figure 2a) and with the theoretical predictions (Table S1 and Figure 2c).

**Figure 3:**
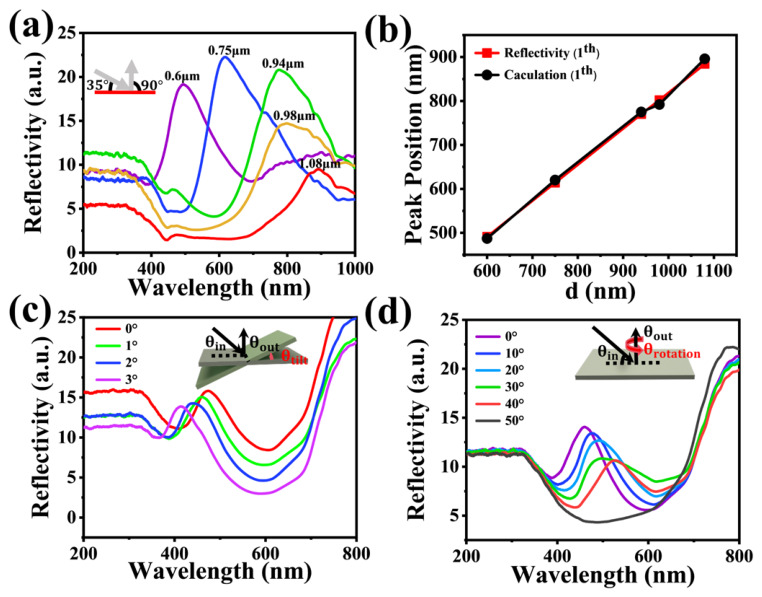
Angle-dependent color-changing properties of WPEs. (**a**) Reflectance spectra of WPEs with different wrinkle periods measured at *θ_in_* = 35° and *θ_out_* = 90°; (**b**) Comparison between experimental and theoretical values of reflective peak positions of WPEs at *θ_in_* = 35°, *θ_out_* = 90°; (**c**) Reflectance spectra of WPEs measured at different tilt angles (*θ_in_* = 35°, *θ_out_* = 90°); (**d**) Reflectance spectra of WPEs measured at different rotation angles (*θ_in_* = 35°, *θ_out_* = 90°), the black lines indicate the paths of incident light and reflected light, the red curved arrow indicates the rotation direction of the film.

In addition, the reflectance spectra of the WPEs with different periods were measured at the same incident angle (35°) and observation angle (90°), as shown in Figure 3a, which agreed well with the theoretically calculated reflection peak positions (Figure 3b). The structural color shifts in the wrinkled PDMS film (d = 550 nm) as a function of tilt angle were also investigated. The reflection peak positions were found to be highly sensitive to changes in tilt angle (Figure 3c). A slight change in tilt angle from 0° to 3° caused the reflection peak to shift from 470 nm to 414 nm. Such tilt-angle-dependent structural color variation of the PDMS films can be well described by the modified Bragg’s law:

(2) $$k\lambda =nd\left(\cos\left(\theta_{in}+\theta_{tilt}\right)-\cos\left(\theta_{out}-\theta_{tilt}\right)\right)$$

where *θ_in_* and *θ_out_* denote the angles between the incident and reflected light and the surface normal, respectively, and *θ_tilt_* represents the tilt angle of the film. According to Equation (7), when the tilt angle increases from 0° to 3°, the theoretical reflection peak is predicted to shift from 450 nm to 389 nm, which is consistent with the experimental results (Table S6). Following the tilt-angle analysis, the rotation angle-dependent structural color behavior of the wrinkled PDMS film (d = 550 nm) was also investigated. As the rotation angle increased from 0° to 40°, the reflection peak redshifted from 470 nm to 535 nm, accompanied by a gradual decrease in peak intensity. When the rotation angle reached 50°, the reflection peak completely disappeared (Figure 3d). The rotation angle-dependent structural color variation of the PDMS films can be predicted by the modified Bragg’s law:

(3) $$k\lambda =nd\left(\cos\theta_{in}-\cos\theta_{out}\right)∕\cos\theta_{rotation}$$

where *θ_rotation_* represents the rotation angle of the PDMS film. According to Equation (8), when the rotation angle increased from 0° to 40°, the theoretical reflection peak was predicted to shift from 451 nm to 588 nm, which was in good agreement with the experimental results (Table S7).

### 2.3. Improvement of Wrinkle Uniformity via Dumbbell-Shaped Substrate Design

**Figure 4:**
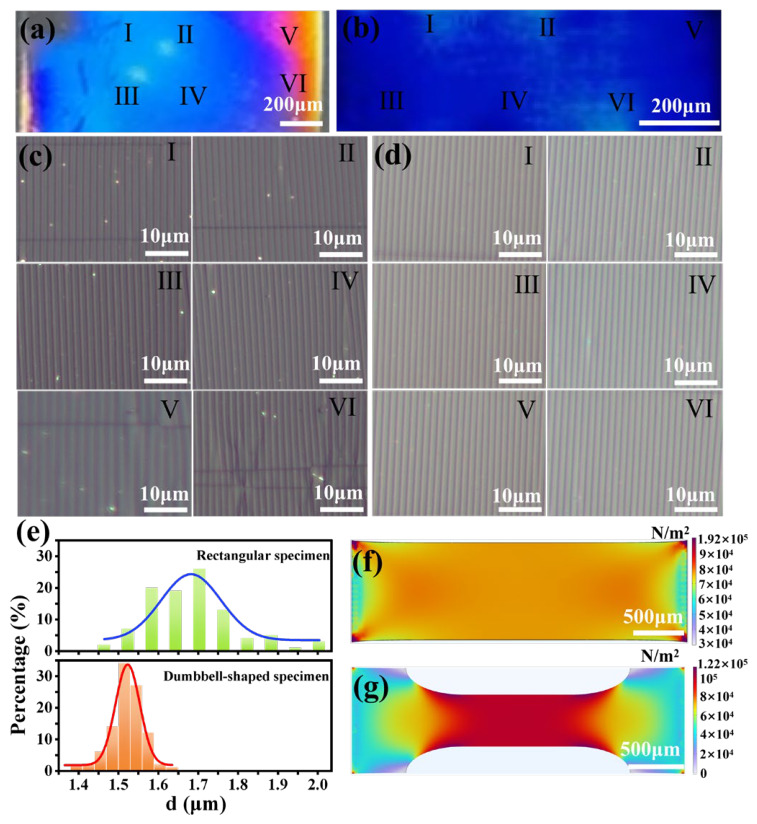
Photographs of WPEs fabricated using a (**a**) rectangular and (**b**) dumbbell-shaped PDMS sample; Optical micrographs of (**c**) rectangular and (**d**) dumbbell-shaped WPEs in different regions. I–VI mark the locations where the optical micrographs were taken; (**e**) Distribution of grating period in different regions of rectangular and dumbbell-shaped specimens, COMSOL stress simulation of (**f**) rectangular and (**g**) dumbbell-shaped PDMS specimens. In (**f**,**g**), the color bars represent the stress distribution (unit: N/m^2^), where blue indicates low stress and red indicates high stress.

The uniformity of wrinkle distributions in the prepared WPEs was further investigated, as shown in Figure 4. For soft, gel-like elastomeric materials, achieving uniform wrinkle patterns across large areas is critical for consistent optical performance. The wrinkles in the central region of the rectangular specimen exhibited good uniformity in both period and orientation (Figure 4a,c(I–IV)), with an average period of approximately 1.68 μm. However, near the clamping ends (Figure 4a,c(V,VI)), both the wrinkle period and alignment varied noticeably, indicating non-uniform deformation across the specimen. To overcome this issue, a dumbbell-shaped specimen with a narrow central region and wider clamping ends was designed and fabricated. The use of non-rectangular specimen geometries to mitigate edge-induced stress concentrations is a well-established strategy in the mechanical testing of soft materials [49]. Inspired by these approaches, we designed a dumbbell-shaped specimen with a narrow central region and wider clamping ends. As shown in Figure 4b,d, the dumbbell-shaped PDMS specimen exhibited a markedly improved uniformity of wrinkle formation. The structural color in Figure 4b appeared continuous and homogeneous across all regions, with an average period of approximately 1.52 μm, indicating a consistent periodic modulation of the surface structure. Consistently, the optical micrographs in Figure 4d (regions I–VI) displayed uniformly aligned wrinkle patterns across the sample, demonstrating that the dumbbell geometry effectively alleviated stress concentrations near the clamping ends and promoted a uniform strain field within the gauge section. Wrinkle periods at 100 systematically selected points were measured from left to right and from top to bottom across the central region of each specimen using optical microscopy. The statistical results are shown in Figure 4e, which present the wrinkle period distributions of the rectangular and dumbbell-shaped specimens with Gaussian fits.

For the rectangular specimen, the wrinkle periods had a standard deviation of 0.1118 μm (CV = 6.64%). In contrast, the dumbbell-shaped specimen showed a smaller deviation of 0.0417 μm (CV = 2.74%), confirming its higher uniformity. Furthermore, COMSOL simulations (COMSOL Multiphysics version 6.2, Stockholm, Sweden) were conducted to analyze the stress distribution under stretching. For the rectangular specimen (Figure 4f), the central region exhibited relatively uniform strain, whereas significant stress concentrations near the clamping ends led to larger wrinkles with irregular orientations, reducing overall uniformity. By contrast, the dumbbell-shaped specimen (Figure 4g) displayed a highly uniform stress field in the narrow central region, with minimal gradients across the measured area. This uniform stress distribution facilitates the formation of wrinkle structures with consistent periods and orientations while effectively mitigating edge-induced stress concentrations. Consequently, the dumbbell-shaped design is more suitable for applications requiring precise optical or mechanical performance.

### 2.4. Patterned Photonic Structures for Multilevel Anti-Counterfeiting Applications

**Figure 5:**
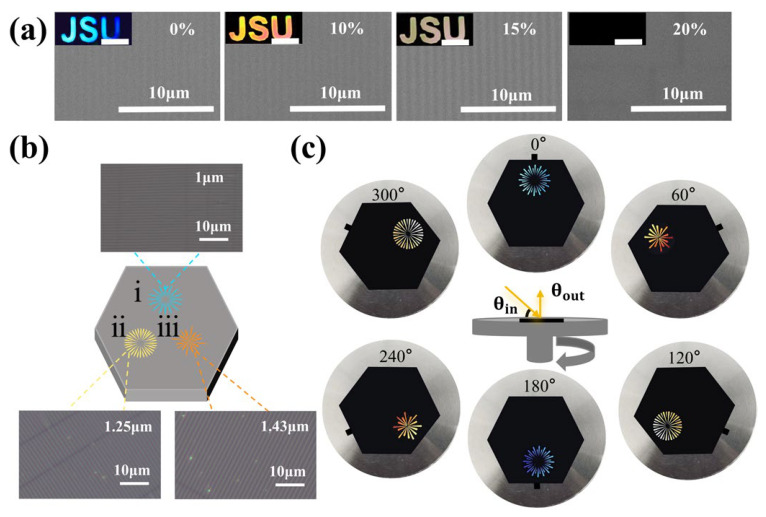
(**a**) SEM images of photonic grafted structures under different tensile strains, the inset photographs show the color-changing behavior of the elastomeric anti-counterfeiting label during stretching (structural color scale bar: 1 cm); (**b**) Schematic illustration of the fabrication process for the dynamic anti-counterfeiting labels. The labels (i), (ii), and (iii) indicate the color development sequence for different pattern shapes; (**c**) Sophisticated dynamic color-shifting security features of the anti-counterfeiting label (*θ_in_* = 45°, *θ_out_* = 90°).

By using custom-designed molds with cut-out patterns during plasma treatment, wrinkled photonic patterns can be easily fabricated on the surface of PDMS films. Wrinkled photonic structures formed exclusively in the exposed regions, producing bright and well-defined structural color patterns. An elastic PDMS wrinkled photonic security label featuring a “JSU” pattern was initially prepared, as shown in Figure 5a. The color of the security label exhibited high sensitivity to mechanical stretching, with significant color shifts occurring even within a small range of stretch ratios (ε). Under a fixed light source and viewing angle, the “JSU” lettering gradually transitioned from blue to yellow as the stretch ratio increased. When the stretch ratio reached the preparation condition’s maximum, the structural color completely disappeared, demonstrating excellent mechanochromic performance with rapid and highly sensitive optical responses. The security label exhibited highly sensitive mechanochromic properties, and its color pattern could be erased at a strain of *ε* = 20% and fully regenerated upon release. The color change was completely reversible, and the grating retained its ordered periodic structure even after 100 loading–unloading cycles (Figure S4). This level of cycling stability is comparable to or better than previously reported mechanochromic photonic elastomers. For example, Lin et al. [38] demonstrated stable structural color in wrinkled photonic elastomers over 1000 cycles, while Zhao et al. [21] reported fatigue in some systems after 50–80 cycles due to interfacial delamination. Our WPEs maintain structural integrity beyond 100 cycles, indicating robust adhesion between the oxidized layer and the PDMS substrate. During the loading and unloading cycles, the color pattern changes are consistent with the reflection peaks (Figure S5). Different periodic gratings were fabricated along three orientations by repeating the S-P-R process with varied cutting modes and stretching directions, yielding periods of 1.0 μm, 1.25 μm, and 1.43 μm. Various functional photonic security labels were subsequently demonstrated (Figure 5b). Under fixed illumination and viewing angles, the pattern became visible when the grating orientation was perpendicular to the incident light, while it became invisible when the grating direction was nearly parallel to the light source. As the rotation angle changed from 0° to 360°, the patterns in the three-axis directions alternately appeared and disappeared, which could be clearly distinguished by the naked eye, offering sophisticated 3D dynamic anti-counterfeiting effects (Figure 5c). This multi-level encryption strategy, combining mechanical strain response and rotational angle dependence, provides two independent modes of optical information control. Recent advances in structural color-based anti-counterfeiting have similarly exploited angle-dependent responses [10,29] and mechanochromism [30,31] for enhanced security. However, unlike static diffraction gratings that show fixed color patterns [25,26], our system integrates both dynamic strain sensitivity and multi-orientation grating design, enabling reversible information erasure and recovery—a feature particularly valuable for next-generation optical security labels [50]. This dynamic and visually distinct optical response underscores the potential of WPE-based systems for advanced 3D anti-counterfeiting applications.

## 3. Conclusions

In summary, wrinkled photonic elastomers (WPEs) with tunable periods and amplitudes, as well as multifunctional photonic anti-counterfeiting labels, were fabricated via a stretch–plasma treatment–release (S-P-R) method. The mismatch in elastic moduli between the rigid surface layer and the underlying elastic substrate, together with the release of interfacial stress, led to the formation of wrinkled photonic structures oriented perpendicular to the pre-strain direction. The periods and amplitudes of the WPEs were precisely controlled by adjusting the plasma power, treatment duration, pre-stretch ratio, and film thickness. The introduction of a dumbbell-shaped geometry effectively improved wrinkle uniformity by mitigating edge-induced stress concentration. Simulations of the stress distribution of a stretched PDMS film explain the formation of relatively uniform wrinkled structures in corresponding regions of the films, providing theoretical guidance for the further optimization of S-P-R methods. Utilizing commercial cut-out stencils, patterned wrinkled photonic security labels were fabricated, exhibiting pronounced angle-dependent color shifts and reversible mechanochromic security features. Sequential processing of the PDMS films under different S-P-R conditions resulted in multi-patterned anti-counterfeiting labels capable of displaying or concealing information at different rotation angles, thereby validating the potential of WPE-based systems for advanced 3D anti-counterfeiting applications. Owing to their gel-like mechanical compliance and optical tunability, the mechanical robustness, optical tunability, and fabrication versatility of these flexible photonic grafting structures hold great promise for applications in anti-counterfeiting, smart windows, wearable devices, display technologies, and mechanical sensing. As a future direction, the well-defined wrinkled PDMS structures demonstrated here can be further exploited as high-fidelity templates to replicate optical functionalities into hydrogel matrices, extending their utility to soft, biocompatible material systems.

## 4. Materials and Methods

### 4.1. Preparation of PDMS 184 Films

Dow Corning Sylgard 184 silicone elastomer (Sylgard 184, Dow Corning, Midland, MI, USA) and curing agent were mixed in a 10:1 weight ratio. Carbon black was added to provide a dark background for structural color observation. The mixture was thoroughly blended, degassed, and then cured at 75°C for 2 h in a specially designed mold.

### 4.2. Preparation of PDMS WPEs and Patterned Devices

Wrinkled photonic elastomers (WPEs) were fabricated using the stretch–plasma oxidation–release (S-P-R) process. The PDMS films were pre-stretched to a desired strain and then subjected to plasma treatment under various conditions. Upon releasing the pre-strain, wrinkles formed on the oxidized rigid surface. Patterned PDMS WPEs were obtained by covering specific regions with cut-out stencils during the plasma oxidation step.

### 4.3. Testing and Characterization

Optical images were captured using an optical microscope (Model: MJ31, Guangzhou Mingmei Optical Technology Co., Ltd., Guangzhou, China), and photographs were taken with a smartphone. Reflectance spectra were recorded using a Shanghai Fuxiang optical spectrometer (EQ 2000, Shanghai Ideaoptics Corp., Ltd., Shanghai, China) equipped with a custom-built adjustable-angle stage to collect reflected light at different viewing angles. The surface morphologies of the wrinkles were examined using field-emission scanning electron microscopy (FE-SEM, FEI Nova Nano450, FEI Company, Hillsboro, OR, USA).

### 4.4. Theoretical Analysis of Wrinkle Geometry

The wrinkle period (d) and amplitude (A) generated via the S-P-R process were further analyzed and theoretically predicted [44] according to the following Equations (4)–(5). These formulas are derived from the classical elastic buckling theory of bilayer systems [44], which is the standard framework for describing wrinkling in stiff-film-on-compliant-substrate systems such as our plasma-oxidized PDMS [45,47].

(4) $$d=\frac{\pi h\left(1+\varepsilon_{applied}\right)}{\left(1+\varepsilon_{pre}\right){\left(1+\varepsilon_{applied}+\zeta \right)}^{\frac{1}{3}}\sqrt{\varepsilon_{c}}}$$

(5) $$A=h\frac{\sqrt{\frac{\varepsilon_{pre}-\varepsilon_{applied}}{\varepsilon_{c}}-1}}{\sqrt{1+\varepsilon_{pre}}{\left(1+\varepsilon_{pre}+\zeta \right)}^{\frac{1}{3}}}$$

(6) $$\zeta =\frac{5\left(\varepsilon_{pre}-\varepsilon_{applied}\right)\left(1+\varepsilon_{pre}\right)}{32}$$

(7) $$\varepsilon_{c}=\frac{1}{4}{\left[\frac{3E_{s}\left(1-\upsilon_{f}^{2}\right)}{E_{f}\left(1-\upsilon_{s}^{2}\right)}\right]}^{\frac{2}{3}}$$

(8) $$\varepsilon =\frac{x_{1}-x_{0}}{x_{0}}\times 100\%$$

where *h* denotes the thickness of the surface film; *E_s_* (2.0 MPa) and *E_f_* (75 GPa) are the elastic moduli of the substrate and surface film, respectively, while υ_s_ (0.48) and υ_f_ (0.27) represent their corresponding Poisson’s ratios. *ε_pre_* is the stretch ratio of the PDMS before plasma oxidation treatment, and *ε_applied_* is the stretch ratio of the PDMS after oxygen plasma treatment. The original length of the PDMS pleated elastomer film prior to stretching is denoted as *X*_0_, and the length after pre-stretching is *X*_1_.

### 4.5. Simulations

The PDMS film model with a length of 1 cm, width of 0.5 cm, and thickness of 1 mm was built in COMSOL Multiphysics. The elastic modulus was set to 2.0 MPa, Poisson’s ratio to 0.48, and density to 970 kg/m^3^. Pre-stretch loading: a displacement load was applied to the other end of the film to achieve a 10% stretch. The stress distribution of the PDMS film under a pre-stretch rate of 10% was analyzed.

## Data Availability

The data that support the findings of this study are available from the corresponding authors upon reasonable request.

## Supplementary Materials

The following supporting information can be downloaded at: https://www.mdpi.com/article/10.3390/gels12050356/s1. Figure S1: (a) Side view of the grating structure with a period of P = 2.49 μm. (b) The periods of PDMS films obtained at different oxygen plasma treatment powers; Figure S2: SEM of PDMS films of different thicknesses. Ɛ = 0.1, P = 300 W, t = 20 s. (a) h = 50 μm; (b) h = 200 μm; (c) h = 500 μm; (d) h = 1000 μm; (e) h = 3000 μm. The periods of PDMS grafting are d_a_ = 1.49 μm, d_b_ = 1.39 μm, d_c_ = 1.28 μm, d_d_ = 1.20 μm, d_e_ = 1.10 μm, respectively; Figure S3: (a) The gratings with different periods reflect colors under the same *θ_in_* and *θ_out_*, (b) Color rendering of the PDMS elastic grating at P = 850 nm at different light source angles at *θ_out_* = 90°; Figure S4: Cycling properties of PDMS films. Number of cycles (a) 0 times, (b) 20 times, (c) 40 times, (d) 60 times, (c) 80 times, (f) 100 times; Figure S5: Structural color change of polydimethylsiloxane (PDMS) film during cyclic stretching (a) initial state and structural color change during stretching, (b) reflectance peaks of thin films under different stretching rates, (c) change in reflection peak position of the film during tensile-release cycle test; Table S1: Theoretical and experimental reflection peaks with a period of 850 nm; Table S2: Theoretical calculation of the reflection peaks with a period of 1400 nm; Table S3: Theoretical calculation of the reflection peaks with a period of 2200 nm; Table S4: Theoretical calculation of the reflection peaks with a period of 2740 nm; Table S5: Theoretical calculation of reflection peaks for PDMS gratings with different periods; Table S6: Theoretical and experimental reflection peaks of PDMS gratings at different tilt angles; Table S7: Theoretical and experimental reflection peaks of PDMS gratings at different rotational angles.

## Abbreviations

PDMS	Polydimethylsiloxane
S-P-R	Stretch–plasma treatment–release
WPEs	Wrinkled photonic elastomers
