# Supplementary material for: Wrinkled Photonic Elastomers with Dynamic Structural Color Patterns for Multilevel Optical Anti-Counterfeiting
Source: Gels. 2026 Apr 23;12(5):356. doi: 10.3390/gels12050356 (PMC13205629; doi:10.3390/gels12050356)
Supplement: Supplementary file 1 [file gels-12-00356-s001.zip › gels-4246758-supplementary.pdf]

Supporting Information for

Wrinkled Photonic Elastomers with Dynamic Structural Color Patterns for Multilevel Optical Anti-Counterfeiting

Xiaoqian Jiang,[†a] Pengjia Yan,[†a] Caiyun Wu,[†a] Junpeng Ke,[a] Wenxiu Hou,[a] Jingran Huang,[a] Zhengzheng Lian,[a] Ting Lü,[*b] and Ling Bai[*a]

School of Materials Science and Engineering, Jiangsu University, Zhenjiang 212013, China

[*]Corresponding authors

E-mail: lingmubai@ujs.edu.cn

[†]These authors contributed equally to this work

Supplementary Results

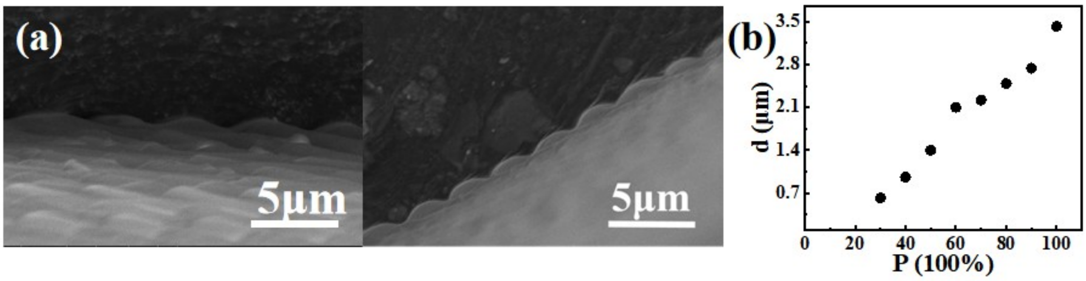

Figure. S1. (a) Side view of the grating structure with a period of P = 2.49 μm. (b) The periods of PDMS films obtained at different oxygen plasma treatment powers.

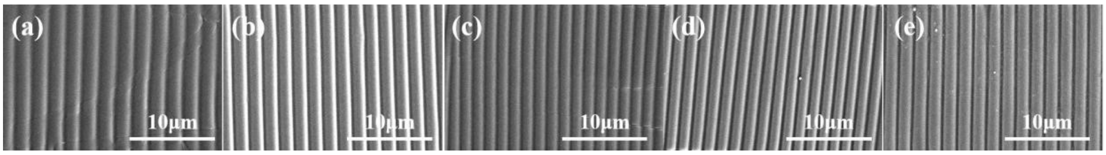

Figure. S2. SEM of PDMS films of different thicknesses. Ɛ = 0.1, P = 300 W, t = 20 s. (a) h = 50 μm; (b) h = 200 μm; (c) h = 500 μm; (d) h = 1000 μm; (e) h = 3000 μm. The periods of PDMS grafting are d_a= 1.49 μm, d_b=1.39 μm, d_c=1.28 μm, d_d=1.20 μm, d_e=1.10 μm, respectively.

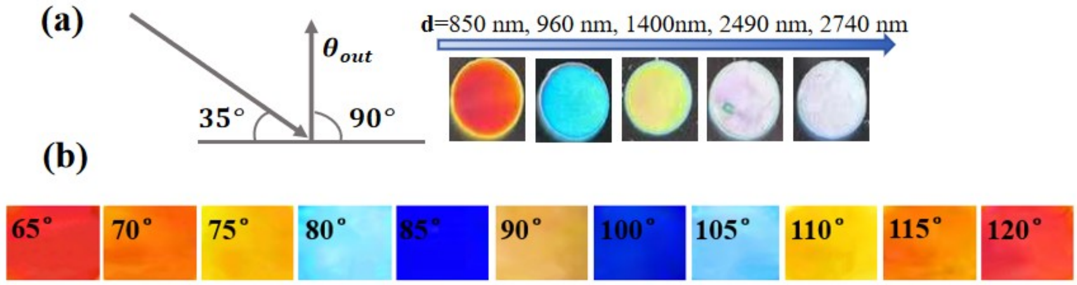

Figure. S3. (a) The gratings with different periods reflect colors under the same θ_{in} and θ_{out}, (b) Color rendering of the PDMS elastic grating at P=850 nm at different light source angles at $\theta_{out} = 90°$.

In the observation mode shown on the left side of Fig. S3a, the grating at 850 nm appears red, the one at 980 nm appears blue, and the one at 1400 nm appears yellow. At 2490 nm and 2740 nm, the primary and secondary diffractions of the grating are shifted to the infrared region, while the tertiary diffraction moves from the ultraviolet to the visible region, resulting in a faint color. The observed color corresponds to the light reflected from the incident illumination.

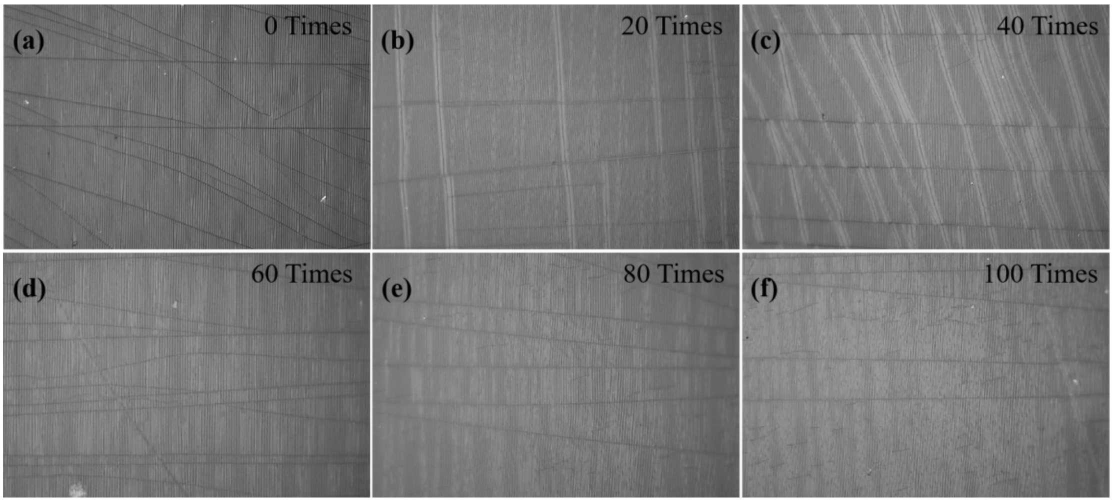

Figure. S4. Cycling properties of PDMS films. Number of cycles (a) 0 times, (b) 20 times, (c) 40 times, (d) 60 times, (c) 80 times, (f) 100 times.

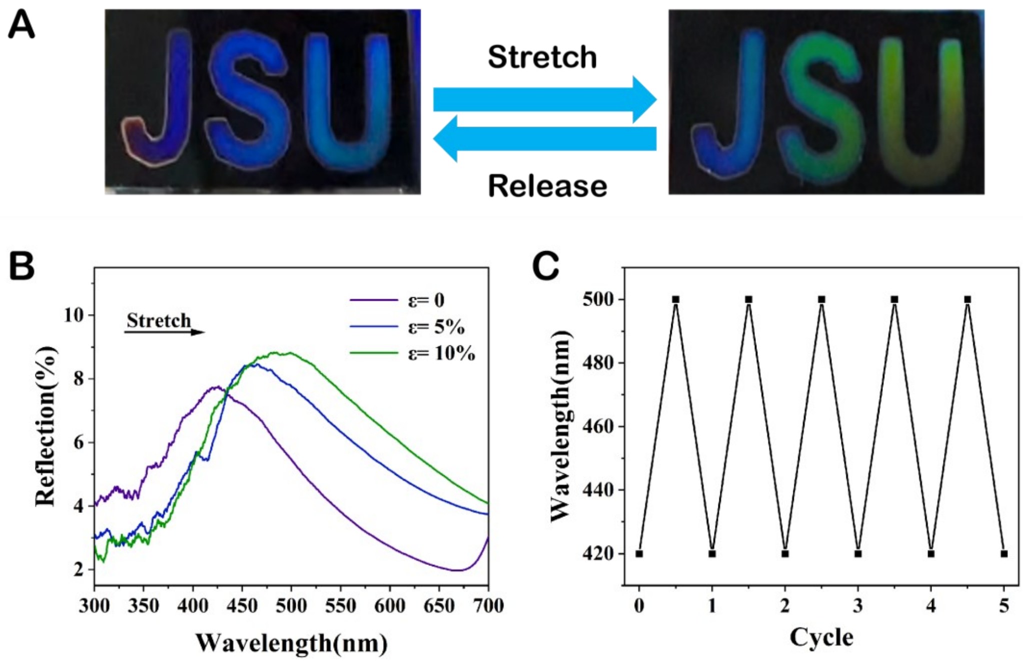

Figure. S5. Structural color change of polydimethylsiloxane (PDMS) film during cyclic stretching (a) initial state and structural color change during stretching, (b) reflectance peaks of thin films under different stretching rates, (c) change in reflection peak position of the film during tensile-release cycle test.

Table S1. Theoretical and experimental reflection peaks with a period of 850 nm.

θ_{out}**(deg)**	**65**	**70**	**75**	**80**	**85**	**90**
Measured value	356	429	504	550	615	658
Calculation(K=1)	337	405	476	548	622	696
Calculation(K=2)	168	202	238	274	311	348

Table S2. Theoretical calculation of the reflection peaks with a period of 1400 nm.

θ_{out}**(deg)**	**60**	**65**	**70**	**75**	**80**	**85**	**90**	**95**
Calculation(K=1)	446	555	667	784	903	1024	1146	1268
Calculation(K=2)	223	277	333	392	451	512	573	634

Table S3. Theoretical calculation of the reflection peaks with a period of 2200 nm**.**

θ_{out}**(deg)**	**60**	**65**	**70**	**75**	**80**	**85**	**90**	**95**
Calculation(K=1)	708	880	1059	1243	1433	1625	1818	2012
Calculation(K=2)	354	440	529	621	716	812	909	1006

Table S4. Theoretical calculation of the reflection peaks with a period of 2740 nm.

**θ_{out}(deg)**	**60**	**65**	**70**	**75**	**80**	**85**	**90**	**95**
Calculation(K=1)	874	1086	1307	1535	1768	2005	2244	2483
Calculation(K=2)	437	543	653	767	884	1002	1122	1241

Table S5. Theoretical calculation of reflection peaks for PDMS gratings with different periods

**d(nm)**	**850**	**960**	**1400**	**2490**	**2740**
Calculation(K=1)	697	804	1148	2041	2246
Calculation(K=2)	348	402	574	1020	1123
Calculation(K=3)	232	268	382	680	748

Table S6. Theoretical and experimental reflection peaks of PDMS gratings at different tilt angles.

**θ_{tilt}(deg)**	**0**	**1**	**2**	**3**
Measured value	470	462	440	414
Calculation(K=1)	450	427	402	389

Table S7. Theoretical and experimental reflection peaks of PDMS gratings at different rotational angles.

**$\theta_{rotation}$(deg)**	**0**	**10**	**20**	**30**	**40**
Measured value	470	475	494	499	535
Calculation(K=1)	451	458	480	520	588
